# Supplementary material for: Explainable Machine Learning Models for Glioma Subtype Classification and Survival Prediction
Source: Cancers (Basel). 2025 Aug 9;17(16):2614. doi: 10.3390/cancers17162614 (PMC12384855; doi:10.3390/cancers17162614)
Supplement: Supplementary file 1 [file cancers-17-02614-s001.zip › cancers-3748134 Supplementary Materials/Supplementary Figures.pdf]

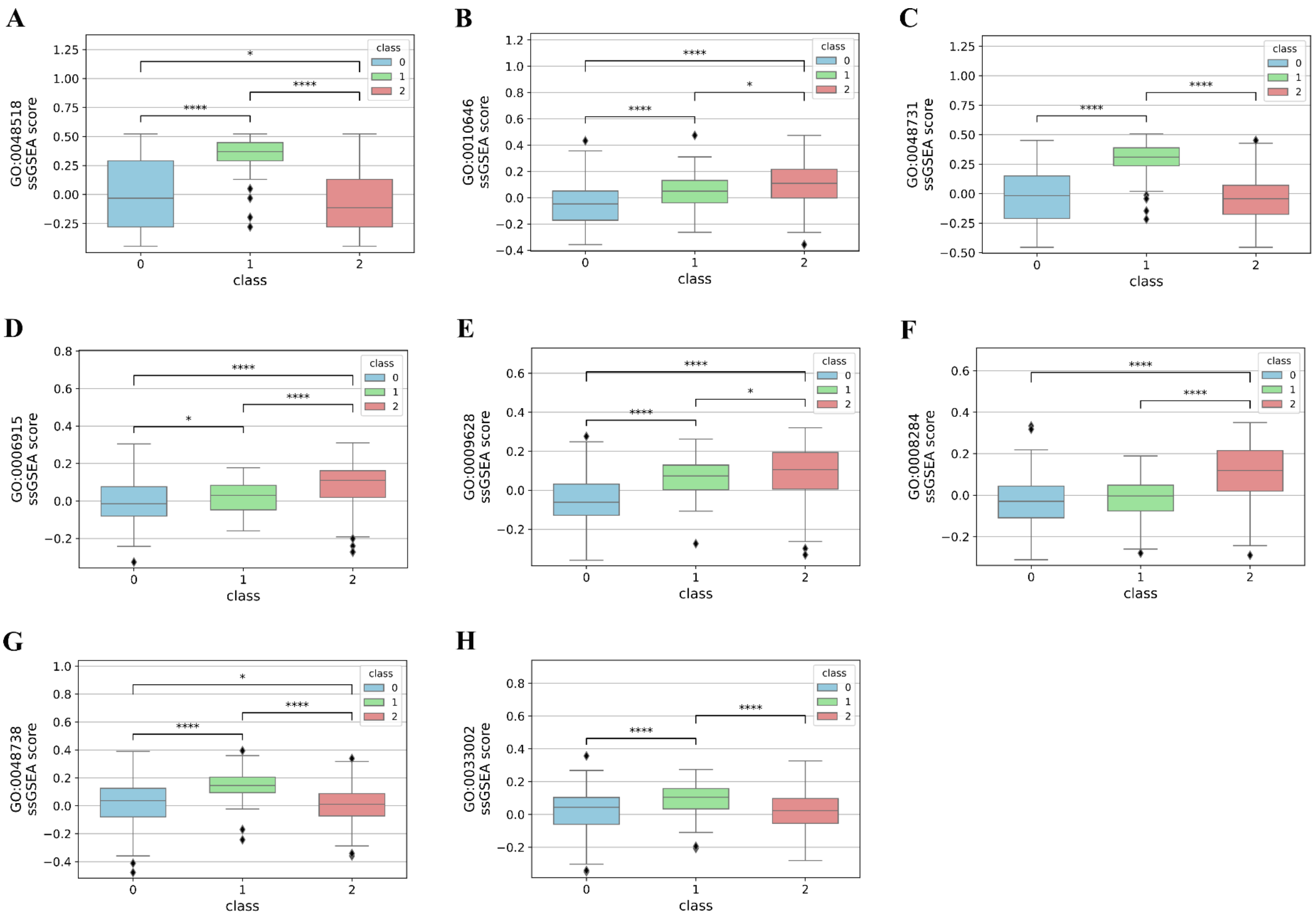

Supplementary Figure S1. Results of the single-sample gene set enrichment analysis (ssGSEA) for Gene Ontology (GO) terms including 13 selected genes. ssGSEA score for (**A**) GO:0048518, (**B**) GO:0010646, (**C**) GO:0048731, (**D**) GO:0006915, (**E**) GO:0009628, (**F**) GO:0008284, (**G**) GO:0048738, (**H**) GO:0033002. Class 0: astrocytoma, class 1: oligodendroglioma, class 2: glioblastoma. Post hoc pairwise Dunn's test: *p<0.05, **p<0.01, ***p<0.001, ****p<0.0001.

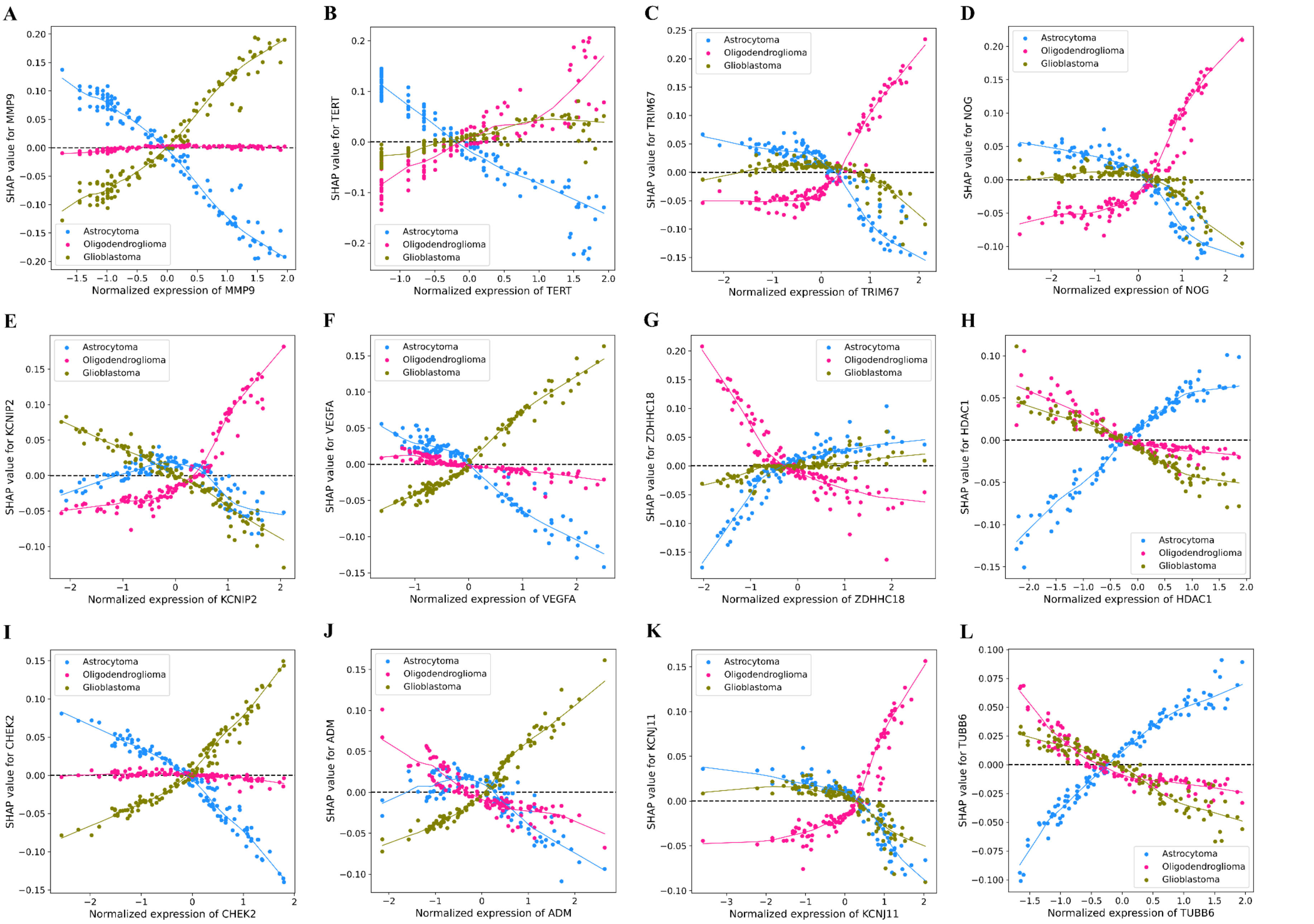

Supplementary Figure S2. Scatterplots between SHAP values and the expression levels of the genes in the classification model for three glioma subtypes: (**A**) MMP9, (**B**) TERT, (**C**) TRIM67, (**D**) NOG, (**E**) KCNIP2, (**F**) VEGFA, (**G**) ZDHHC18, (**H**) HDAC1, (**I**) CHEK2, (**J**) ADM, (**K**) KCNJ11, (**L**) TUBB6.

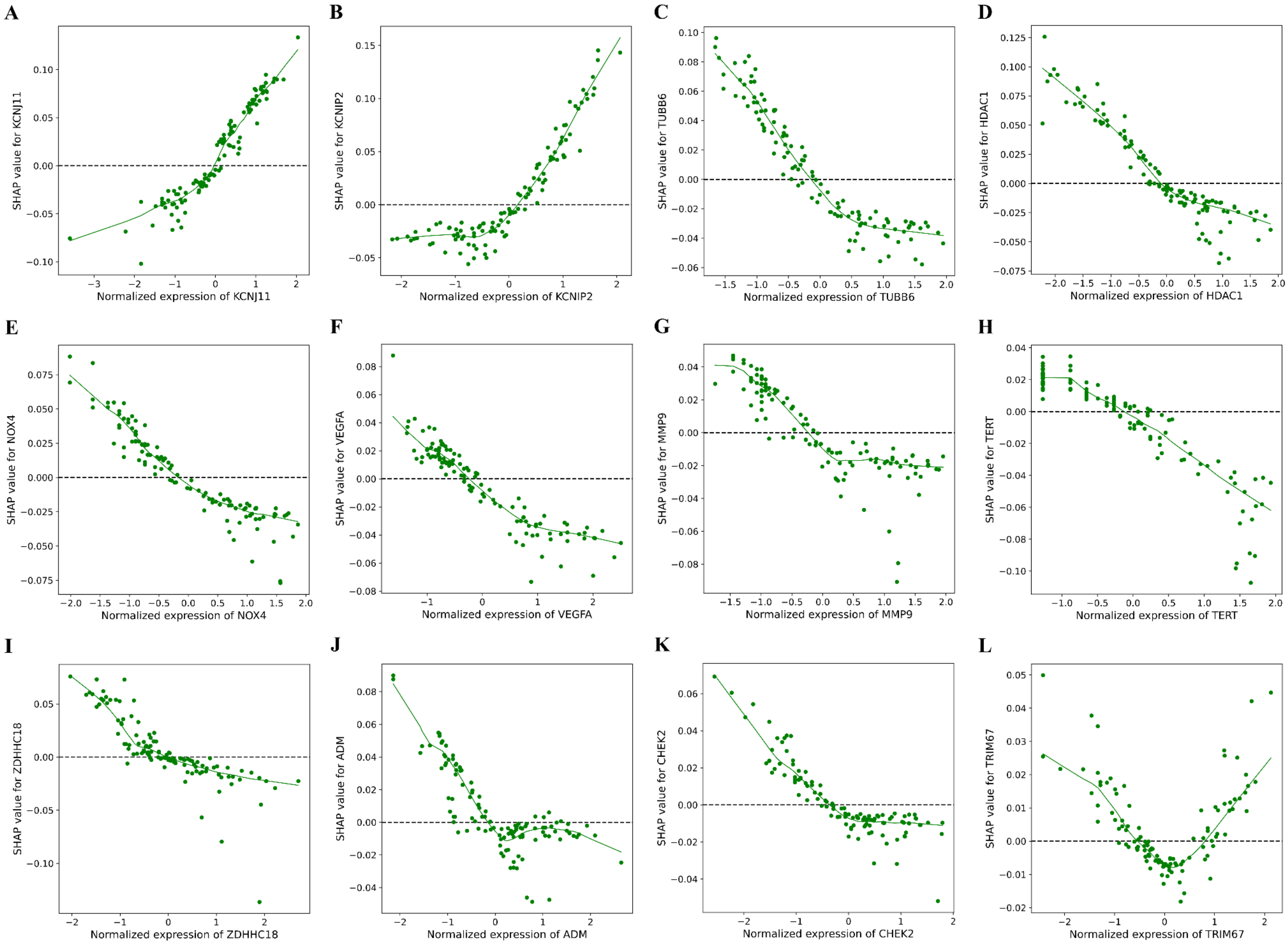

Supplementary Figure S3. Scatterplots between SHAP values and the expression levels of the genes in the model for predicting overall survival of patients with glioma: (**A**) KCNJ11, (**B**) KCNIP2, (**C**) TUBB6, (**D**) HDAC1, (**E**) NOX4, (**F**) VEGFA, (**G**) MMP9, (**H**) TERT, (**I**) ZDHHC18, (**J**) ADM, (**K**) CHEK2, (**L**) TRIM67.
